# News and Coming Events

**Published:** 1907-03-23

**Authors:** 


### NEWS AND COMING EVENTS.

H.R.H. Princess Christian of Schleswig-Holstein has consented to become patroness of the Loughborough Hospital.

A "suggestion box" has been placed in the Haddo House Cottage Hospital for the benefit of visitors. Cards containing written suggestions can be placed in the box, and will be laid before the Governors for consideration.

The Annual Court of Governors of the Mount Vernon Hospital for Consumption will be held at the offices, No. 7 Fitzroy Square, W., on Wednesday, March 27, at four o'clock, the Marquis of Zetland, K.T., President, in the chair.

Hospitals in the county of London, or within seven miles of Charing Cross, desiring to participate in the grants made by King Edward's Hospital Fund for the year 1907, must make application before March 31 to the Honorary Secretaries, 81 Cheapside, E.C.

The annual meeting of the Royal Hospital for Diseases of the Chest, held on March 15, calls attention once more to the urgent necessity for the removal of this hospital to a more suitable and better site. Certainly the City Road is the last place to select for a consumption hospital, having regard to modern requirements. The admission of cardiac and other medical cases does not really affect the question, which is so urgent that it ought to be faced and met by the Governors without further delay.

We congratulate the authorities of the Essex and Colchester Hospital on the improvements they have introduced into the annual report. The accounts are now kept upon a uniform system, the statistical information is clearly and exhaustively given, the contributions from the country districts are properly classified, and the list of annual subscribers is made to agree with the audited accounts. Facts like these should win public confidence and bring revenue in sufficient quantity to the hospital coffers.

At the annual meeting of the St. Cross Hospital, Rugby, the following rule was proposed and unanimously adopted: "No person shall attend as an out-patient (unless under exceptional circumstances), whose average weekly income exceeds the following scale: One person in family, 18s. per week; two persons in family, 21s. per week; three persons in family, 24s. per week; and so on—3s. for each additional member of the family." In the course of the discussion, Mr. Walton pointed out that the alteration would not curtail but extend the benefits of the hospital.

At the annual meeting of the Bedford County Hospital two important points were brought up for discussion. The first was the matter of the abuse of the out-patient department. Thirty years ago, said Dr. Kinsey, in calling attention to the matter, there were 2,000 out-patients, but last year the number had grown to 19,000, and he thought that a committee should be appointed to inquire into the whole matter. The poor attached too much value to medicine, whereas they ought to be taught the value of fresh air, cleanliness, and proper food. The second matter was the proposal to elect lady members on the board of management, a proposal which evoked keen discussion, several of the Governors expressing themselves strongly against it. The motion that it would be desirable to have two lady members on the board was finally lost by 30 votes against 21, several of the Governors not voting.

During the past year the Belfast Samaritan Hospital has been thoroughly renovated at a total cost of £356 12s. 6d.

"The Midwives' Record and the Monthly Nurse" is a new journal for certified midwives and maternity nurses, published by Messrs. Spottiswoode and Company, Limited. The second number, though small, is well got up, and contains some interesting reading matter.

The next meeting of the Therapeutical Society will be held at the Apothecaries' Hall, Blackfriars, E.C., on Tuesday, March 26, at 4.30 p.m. The following papers will be read: Dr. W. E. Dixon, "How Drugs produce their Effects"; Dr. Nestor Tirard, "Opium in the Treatment of Pneumonia."

Mr. J. W. Garnier has been appointed steward of the *Dreadnought* Hospital, Greenwich, vice Mr. J. G. Buckle, B.A., promoted to be assistant secretary. There were a large number of applicants, including several nominated by the Cambridge Appointments Committee and the Officers' Employment Bureau. Six very excellent candidates appeared before the Board, and Mr. Garnier was finally elected.

At the monthly committee of the London Branch of the Children's Home and Orphanage it was reported that an old boy of the Home had obtained an appointment as headmaster of an important school in Shanghai. Ten boys and five girls were passed for admission. The number now resident in the various branches of this institution is about 1,750. The income shows a slight advance on last year's, but an increase of £2,000 is needed by March 31 if the growing expenditure is to be met.

The Gemeenteraad of Amsterdam has decided to institute an official medical inspection of secondary schools, and has appointed twelve local practitioners—among them one lady doctor—as official inspectors. The inspectors will commence their duties in May, and are authorised to report fully on the hygienic and sanitary conditions of the schools, and also to report on such children as in their opinion are suffering from affections such as adenoids, caries of the spine, and auditory or visual disturbances, etc.

At the annual meeting of the Ulster Hospital for Women and Children, the Chairman, the Earl of Shaftesbury, stated that the inadequacy of the present building having been increasingly manifested during the year, a small committee had been formed to take steps for procuring the funds necessary for erecting a new hospital. The valuable and excellently situated ground at Templemore Avenue owned by the Board is abundantly large enough to allow of this work being accomplished without interference with the old building. Mr. G. W. Wolff, M.P., was approached, and very generously and promptly opened the list with a promise of £1,000. A condition attached, that the whole of the money needed should be secured before commencing work, being quite in agreement with the Board's decision, was gladly accepted. Other and liberal donations have been promised, and the governing body of the hospital appeals with great earnestness to all friends of suffering children and women to supply the sum (estimated at £10,000) which will be needed. The Ulster Hospital for Children and Women supplies the wants of a general hospital in a large working-class population of over 100,000. The hospital is fortunate in possessing the freehold of the ground upon which it stands, and there is ample room for extension of the present limited and overcrowded accommodation.

